# Explainable automated pain recognition in cats

**DOI:** 10.1038/s41598-023-35846-6

**Published:** 2023-06-02

**Authors:** Marcelo Feighelstein, Lea Henze, Sebastian Meller, Ilan Shimshoni, Ben Hermoni, Michael Berko, Friederike Twele, Alexandra Schütter, Nora Dorn, Sabine Kästner, Lauren Finka, Stelio P. L. Luna, Daniel S. Mills, Holger A. Volk, Anna Zamansky

**Affiliations:** 1 Information Systems Department, University of Haifa, Haifa, Israel; 2 Faculty of Electrical Engineering, Technion, Israel Institute of Technology, Haifa, Israel; 3 Department of Small Animal Medicine and Surgery, University of Veterinary Medicine Hannover, Hannover, Germany; 4 Cats Protection, National Cat Centre, Chelwood Gate, Sussex UK; 5 School of Veterinary Medicine and Animal Science, São Paulo State University (Unesp), São Paulo, Brazil; 6 School of Life Sciences, Joseph Bank Laboratories, University of Lincoln, Lincoln, UK

**Keywords:** Computer science, Animal behaviour, Pain

## Abstract

Manual tools for pain assessment from facial expressions have been suggested and validated for several animal species. However, facial expression analysis performed by humans is prone to subjectivity and bias, and in many cases also requires special expertise and training. This has led to an increasing body of work on automated pain recognition, which has been addressed for several species, including cats. Even for experts, cats are a notoriously challenging species for pain assessment. A previous study compared two approaches to automated ‘pain’/‘no pain’ classification from cat facial images: a deep learning approach, and an approach based on manually annotated geometric landmarks, reaching comparable accuracy results. However, the study included a very homogeneous dataset of cats and thus further research to study generalizability of pain recognition to more realistic settings is required. This study addresses the question of whether AI models can classify ‘pain’/‘no pain’ in cats in a more realistic (multi-breed, multi-sex) setting using a more heterogeneous and thus potentially ‘noisy’ dataset of 84 client-owned cats. Cats were a convenience sample presented to the Department of Small Animal Medicine and Surgery of the University of Veterinary Medicine Hannover and included individuals of different breeds, ages, sex, and with varying medical conditions/medical histories. Cats were scored by veterinary experts using the Glasgow composite measure pain scale in combination with the well-documented and comprehensive clinical history of those patients; the scoring was then used for training AI models using two different approaches. We show that in this context the landmark-based approach performs better, reaching accuracy above 77% in pain detection as opposed to only above 65% reached by the deep learning approach. Furthermore, we investigated the explainability of such machine recognition in terms of identifying facial features that are important for the machine, revealing that the region of nose and mouth seems more important for machine pain classification, while the region of ears is less important, with these findings being consistent across the models and techniques studied here.

## Introduction

According to the International Association for the Study of Pain (IASP), pain is an “unpleasant sensory and emotional experience associated with, or resembling that associated with, actual or potential tissue damage”^1^. It is particularly important to recognize that “verbal description is only one of several behaviors to express pain; inability to communicate does not negate the possibility that a human or a nonhuman animal experiences pain”. However, in the absence of verbal indications from patients, the accurate assessment of an individual’s pain relies upon the inferences made by clinicians. Given the lack of standardised and objectively applicable tools to assess pain in such contexts^2^, this process is inherently challenging and a ubiquitous problem regarding non-human animals due to their non-verbal status^3^. Surveys in the veterinary profession clearly indicate that the lack of such tools may well interfere with an accurate assessment and classification and thus appropriate treatment of pain. For instance, a study of attitudes and beliefs of Queensland veterinarians in relation to postoperative pain and preoperative analgesia in dogs revealed that nearly one-fifth of respondents doubted their confidence in their knowledge about post surgical pain; 42% acknowledged difficulties recognising pain, and nearly one-quarter were unsure or negative about the capacity of veterinarians to recognise pain^4^. These findings were also supported in a study investigating the attitudes of veterinary practitioners in New Zealand to pain and analgesia in cats and dogs, where only 58% of respondents considered their knowledge in the area of assessment and treatment of pain to be adequate^5^. Another study of UK veterinarian’s attitudes to chronic pain in dogs identified difficulties with pain assessment as a major barrier to adequate treatment of chronic pain^6^.

Despite the inherent challenges of pain assessment in non-human animals, species-specific pain scales which focus on changes in animal’s facial features such as their expressions can provide useful practical instruments for proxy pain assessment. A decade ago, the first grimace scales were developed for rodents and similar scales are now validated for many mammalian species^7^, including rats^8^, rabbits^9^, horses^10^, pigs^11^, sheep^12^, ferrets^13^ and cats^14,15^.

Cats are one of the most challenging species in the context of pain assessment and management due to a variety of factors, including reduced physiological tolerance and adverse effects to common veterinary analgesics^16^, a lack of strong consensus over key behavioural pain indicators^17^ and human limitations in accurately interpreting feline facial expressions^18^. These factors may contribute to cats being prescribed less analgesic drugs by veterinarians compared to dogs, even when the predicted degree of pain experienced between both species is similar^19–21^.

Three different manual pain assessment scales have been developed and validated in English for domestic cats: the UNESP-Botucatu multidimensional composite pain scale (MCPS)^22^, the Glasgow composite measure pain scale (CMPS),^23^ and the Feline Grimace Scale (FGS)^15^. The latter was further used for a comparative study in which human’s assignment of FGS to cats during real time observations and then subsequent FGS scoring of the same cats from still images were compared. It was shown that there was no significant difference between the scoring methods^24^, indicating images can be a reliable medium from which to assess pain, compared to direct, real-time observations.

However, even though there is a good demonstrated agreement between FGS scorers with different experiences and backgrounds^25^, there are potentially other less explored factors that might influence the reliability and validity of these types of manual scoring methods that rely on the subjective judgements of humans. This leads to the need for the development of more objective methods for scoring and assessing pain which are less susceptible to human bias. A step in this direction was taken by Finka and colleagues^26^, who used geometric landmarks to identify and quantify facial shape changes associated with pain. Images of 29 domestic short-haired female cats undergoing ovariohysterectomy were (reliably) manually annotated using 48 landmarks specifically chosen for their relationship with underlying facial musculature and their relevance to cat-specific facial action units. A significant relationship was found between pain-linked Principal Components related to facial shape variation and the UNESP-Botucatu MCPS tool^22^.

These results served as a starting point for our previous exploration of automated detection of pain in cats^27^, where two different approaches were compared: a manually annotated facial landmark-based (e.g.^26^) approach and a deep learning approach. While both approaches reached comparable accuracy of approximately 72%, a significant limitation was that the study population was highly homogenous, limited to young, adult female cats of a single breed and submitted to only one type of postoperative pain condition.

Factors such as breed^28,29^, age^30^ and potentially their interaction^31^, as well as sex^32^ and particularly neuter status in adult males (e.g.^33^) may all affect craniofacial morphology in cats, and thus potentially the nature of pain-related facial information extractable from associated images. Establishing generalizability of our developed approaches^26,27^ with broader cat characteristics across more heterogeneous populations is therefore a crucial step on the path towards accurate automated cat pain recognition.

The contribution of this study is twofold, addressing the following research questions:

1. *To what extent can a machine recognize pain in cats in a more naturalistic or ‘noisy’ population (e.g. variations in breed, sex and painful conditions)?* We address this question by repeating and expanding the scope of the comparative study outlined in^27^ using two approaches to the automatization of cat pain recognition (landmark-based and deep learning based) on a new dataset of 84 client-owned cats presented to the Department of Small Animal Medicine and Surgery of the University of Veterinary Medicine Hannover. Different breeds with varying age, sex, and medical history were included; the cats were also scored using the Glasgow composite measure pain scale (CMPS) by veterinarians to provide an indication of degree to which pain was present using this previously validated behaviour based tool.
2. *Which facial features are most important for the machine in relation to pain recognition performance?* We address this question by using explainable AI (XAI^34^) methods to investigate the roles played by different cat face regions: ears, eyes, mouth, and nose in machine pain recognition.

## Results

For narrative purposes we preface our results with essential and practical aspects to improve understanding for those less familiar with AI methods, presenting a high-level overview of the used approaches, as well as with the dataset description.

### Overview

**Figure 1:**
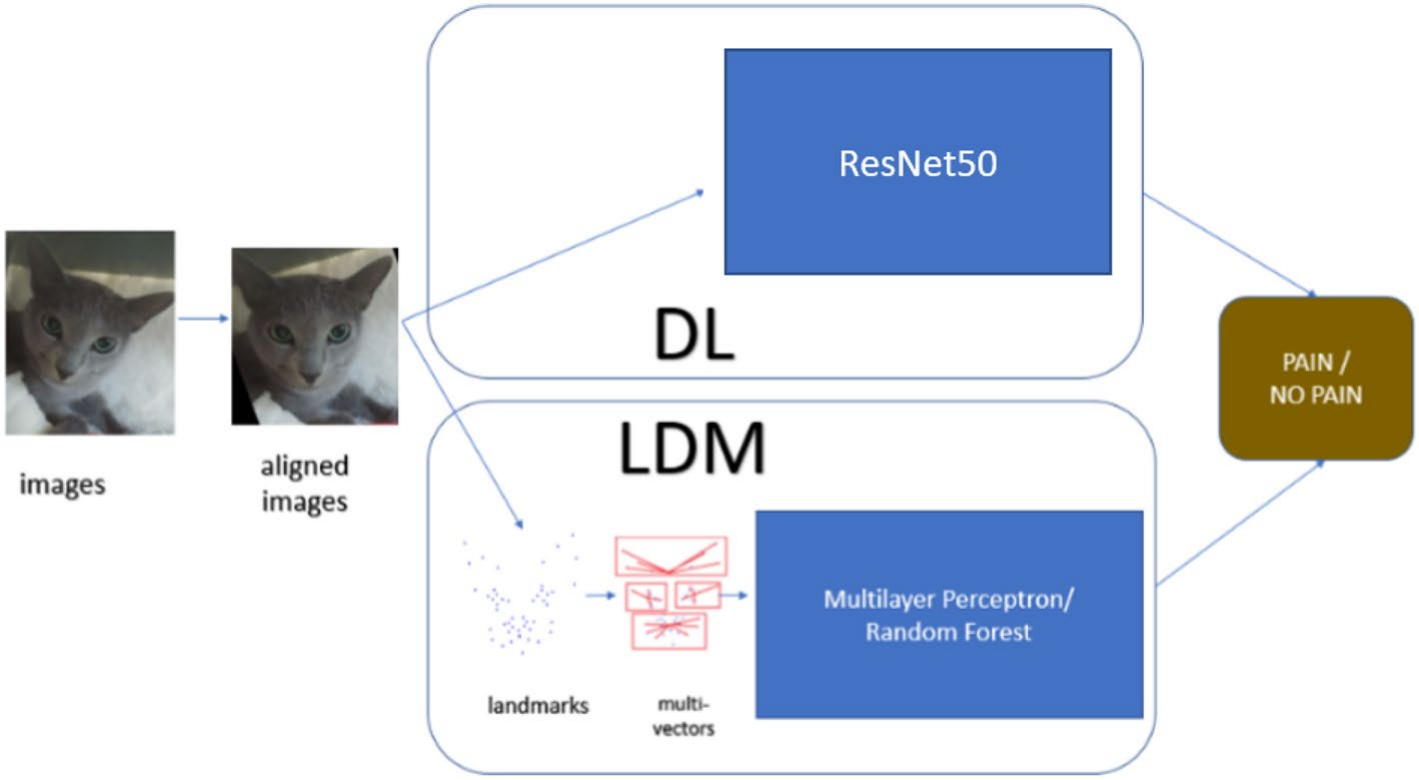
High-level overview of the comparative study: deep learning (DL) and landmark-based (LDM) approaches.

Figure 1 presents a high-level overview of the two pipelines for the deep learning (DL) and landmark-based (LDM) approaches used in this study. Both of the pipelines start with cat facial alignment, using the method described in Feighelstein et al.^27^, which is based on manual landmark annotations. The aligned images are then fed to the deep learning models as is, while the landmark-based approach uses the XY locations of the 48 landmarks, which serve as cat face “abstractions”. These landmarks are then used to create multi-vectors according to cat facial regions capturing ears, nose/mouth, eyes, as described in Feighelstein et al.^27^. These vectors form the final input to the machine learning models (Multilayer Perceptron and Random Forest are used here).

### Dataset

Owners provided written informed consent to provide data that can be used for research, regulated by the law and regulations for research in Lower Saxony, Germany. All experiments were performed in accordance with relevant guidelines and regulations. The current protocol was reviewed and approved by the Ethical Committee of the Medical University of Hannover; the Ethical Committee of the University of Haifa waived ethical approval.

**Figure 2:**
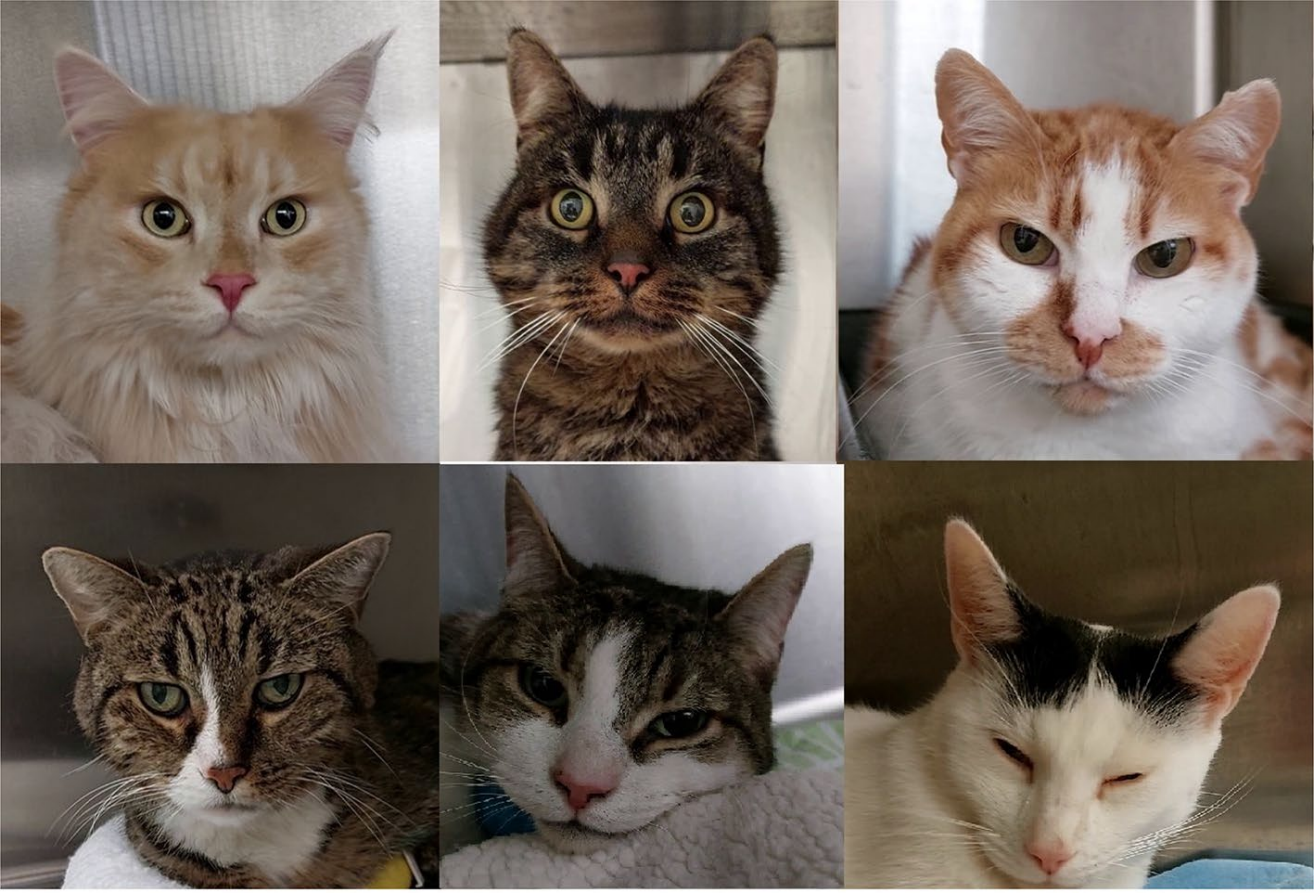
Example images. Top row: cats from Class 2 (‘no pain’) (CMPS scoring $<4$ and no reason to suspect pain); Bottom row: cats from Class 1 (‘pain’) (CMPS scoring $\ge 5$ and clinical reason for pain).

Our dataset included images of 84 client-owned cats presented between May 2021 and April 2022 to the Department of Small Animal Medicine and Surgery of the University of Veterinary Medicine Hannover. Cats were recorded in a cage, where they were free to move (and hide themselves), having also free access to water and food during the whole hospitalisation period, as well as to a litter box inside their cage. The cats were captured using a mobile phone video recorder using a self-developed app, from which the best frames (recording distance approximately 10 cm with cat facing camera) were extracted. Example images are presented on Figure 2. Any presented cat was in principle eligible for the study. Cats of different breeds, ages, sex, and medical history were included. Brachycephalic cats, who have an extreme facial conformity (compared to mesocephalic cats), as well as cats with facial wounds or patients with neurological diseases that affect the facial expression were excluded.

The cats were scored during clinical examination using the CMPS-feline instrument^23^ in their cage at least half an hour after the last clinical examination, in order to enable a rest period and to reduce scoring bias. The CMPS-feline instrument includes seven categories, referring to changes in the cat’s behavior as well as in the cat’s face. A total maximum of 20 points is possible, with scores $\ge$5 considered an intervention threshold^23^. In this study, the images were divided into two classes henceforth referred to as ‘pain’ and ‘no pain’. Cats with CMPS scores of 4 were excluded to allow a clearer distinction between ‘pain’ and ‘no pain’ classes. Moreover, cats with CMPS scores of $\ge$5 which had no clinical reason to suspect pain were also excluded. This led to Class 1 (‘pain’) including 42 cats satisfying the following two conditions: (i) with CMPS scores of $\ge$5, and (ii) with clinical reasons to suspect pain. The clinical reasons for suspected pain of the cats in Class 1 are listed in Table 2. The most frequent reasons for presentation were various bone fractures (e.g. of the femur, pelvis or humerus), followed by gastrointestinal foreign bodies and surgery and problems concerning the urinary tract. Class 2 (i.e. ‘no pain’) was balanced with 42 cats using random undersampling and included cats who satisfied the following two conditions: (i) CMPS scores of <4, and (ii) with no known clinical reason to suspect pain. Only one sample frame of an individual was included in each of the two classes (see Fig. 4).

Tables 1 and 2 present the list of the participants in the two classes, presenting demographic information including sex, neuter status, breed, age and clinical condition which was the reason for presentation at the clinic.

**Table 1:** Participant demographics - class ‘no pain’. *CMPS scoring performed at check-up examination after recovery and/or successful treatment.

Id	Sex	Neutered	Breed	Age	Clinical condition	CMPS score	Pain?
1	m	Yes	European short haired cat	18 months	Foreign body	2	No*
2	f	Yes	European short haired cat	92 months	Anemia	0	No
3	m	Yes	Maine Coone	22 months	Azotemia	1	No
4	m	Yes	European short haired cat	58 months	Anemia	1	No
5	f	Yes	European short haired cat	96 months	Anemia	2	No
5	m	Yes	British short haired cat	164 months	Intoxication	2	No
7	m	Yes	British short haired cat	68 months	Anemia	2	No
8	m	Yes	British short haired cat	20 months	Pneumothorax	0	No*
9	m	No	British long haired cat	7 months	Paraneoplastic syndrome	2	No
10	m	Yes	Maine Coone	84 months	Otitis externa	1	No
11	f	Yes	European short haired cat	15 months	Seizures	1	No
12	m	No	British short haired cat	8 months	Paraparesis	0	No
13	m	Yes	European short haired cat	152 months	Hyphema	0	No
14	m	Yes	Sibirian forest cat	10 months	Vomiting	0	No
15	m	Yes	European short haired cat	40 months	Diaphragmatic rupture	1	No*
16	m	Yes	European short haired cat	19 months	Ataxia	2	No
17	f	Yes	European short haired cat	163 months	Vomiting	2	No
18	m	Yes	European short haired cat	156 months	Corneal ulcer	0	No
19	f	Yes	Maine Coone	69 months	Anemia	2	No
20	m	No	Maine Coone	58 months	lack of appetite	3	No
21	f	No	European short haired cat	unknown	Fracture of the humerus	1	No*
22	m	Yes	European short haired cat	139 months	Avulsion of the tail	0	No*
23	m	Yes	Ragdoll	55 months	Pulmonary edema	0	No
24	m	Yes	Maine Coone	29 months	Intestinal invagination	1	No
25	m	Yes	European short haired cat	79 months	Vomiting	0	No
26	m	Yes	Bengal cat	9 months	Vomiting	1	No
27	m	Yes	Ragdoll	115 months	Anemia	2	No
28	m	No	British short haired cat	72 months	Pulmonary edema	2	No
29	m	Yes	European short haired cat	56 months	Vomiting	0	No
30	f	Yes	Russian blue cat	165 months	Planned surgery on the eye	0	No*
31	f	Yes	Exotic short haired cat	67 months	Anemia	0	No
32	f	Yes	Norwegian forest cat	140 months	Intestinal tumor	2	No*
33	m	Yes	European short haired cat	168 months	Seizures	0	No
34	m	Yes	Maine Coone	46 months	Pleural effusion	1	No
35	f	Yes	European short haired cat	192 months	Pleural effusion	0	No
36	f	Yes	European short haired cat	103 months	Vomiting	0	No
37	m	Yes	British short haired cat	45 months	Enteral foreign body	3	No*
38	f	No	British short haired cat	8 months	Lymphoma	1	No
39	m	Yes	European short haired cat	19 months	Lymphoma	0	No
40	m	Yes	European short haired cat	19 months	Cystitis	2	No*
41	f	Yes	European short haired cat	134 months	Dyspnea	1	No
42	m	Yes	European short haired cat	113 months	Anemia	2	No

**Table 2:** Participant demographics - class ‘pain’.

Id	Sex	Neutered	Breed	Age	Clinical condition	CMPS Score	Pain?
43	f	Yes	Birman cat	105 months	Pyometra	5	Yes
44	f	No	Siamese cat	24 months	Fracture of the femur	5	Yes
45	m	Yes	British short haired cat	15 months	Dislocation of the tarsal joint	6	Yes
46	f	Yes	Russian blue cat	101 months	Monoparesis	8	Yes
47	f	Yes	European short haired cat	130 months	Polytrauma	10	Yes
48	f	Yes	British short haired cat	19 months	Coprostasis	9	Yes
49	f	Yes	European short haired cat	156 months	Urolithiasis	5	Yes
50	m	Yes	European short haired cat	36 months	Cholecystopathy	7	Yes
51	m	Yes	British short haired cat	72 months	FLUTD	5	Yes
52	m	Yes	European short haired cat	19 months	Trauma after car accident	7	Yes
53	m	Yes	British short haired cat	27 months	Avulsion of the tail	5	Yes
54	f	Yes	European short haired cat	51 months	Pelvic fracture	5	Yes
55	m	Yes	European short haired cat	120 months	Lower jaw symphysiolysis	8	Yes
56	m	Yes	Russian blue cat	61 months	Pelvic fracture	8	Yes
57	m	Yes	European short haired cat	19 months	FLUTD	8	Yes
58	m	Yes	European short haired cat	144 months	Pleuroperitoneal hernia	6	Yes
59	m	Yes	British short haired cat	86 months	Fracture of the humerus	5	Yes
60	f	No	European short haired cat	unknown	Avulsion of the tail	7	Yes
61	m	Yes	European short haired cat	115 months	Intestinal invagination	5	Yes
62	m	Yes	Maine Coone	29 months	Polytrauma	5	Yes
63	m	Yes	European short haired cat	60 months	Corneal ulcer	7	Yes
64	m	Yes	European short haired cat	56 months	Pelvic fracture	6	Yes
65	m	Yes	British short haired cat	32 months	Fracture of the femur	6	Yes
66	f	Yes	Norwegian forest cat	18 months	Fracture of the femur	7	Yes
67	f	Yes	Norwegian forest cat	18 months	FLUTD	5	Yes
68	m	Yes	European short haired cat	88 months	FLUTD	5	Yes
69	m	Yes	European short haired cat	31 months	Fracture of radius and ulna	6	Yes
70	m	Yes	Bengal cat	67 months	Pelvic fracture	7	Yes
71	m	Yes	European short haired cat	18 months	Lameness	5	Yes
72	f	Yes	European short haired cat	74 months	Polytrauma	5	Yes
73	m	Yes	European short haired cat	132 months	FLUTD	6	Yes
74	m	Yes	European short haired cat	168 months	Meningoencephalytis	7	Yes
75	m	No	British long haired cat	7 months	Coprostasis	5	Yes
76	m	Yes	Sibirian forest cat	26 months	Pancreatitis	5	Yes
77	m	Yes	European short haired cat	127 months	Pelvic fracture	6	Yes
78	f	No	European short haired cat	14 months	Pelvic fracture	10	Yes
79	f	No	European short haired cat	10 months	Fracture of the femur	5	Yes
80	f	Yes	European short haired cat	60 months	Fracture of the femur	5	Yes
81	f	Yes	European short haired cat	124 months	Penis necrosis	7	Yes
82	m	Yes	European short haired cat	50 months	Peritonitis	11	Yes
83	f	Yes	European short haired cat	141 months	Fracture of the femur	7	Yes
84	m	No	European short haired cat	16 months	Polytrauma	5	Yes

**Figure 3:**
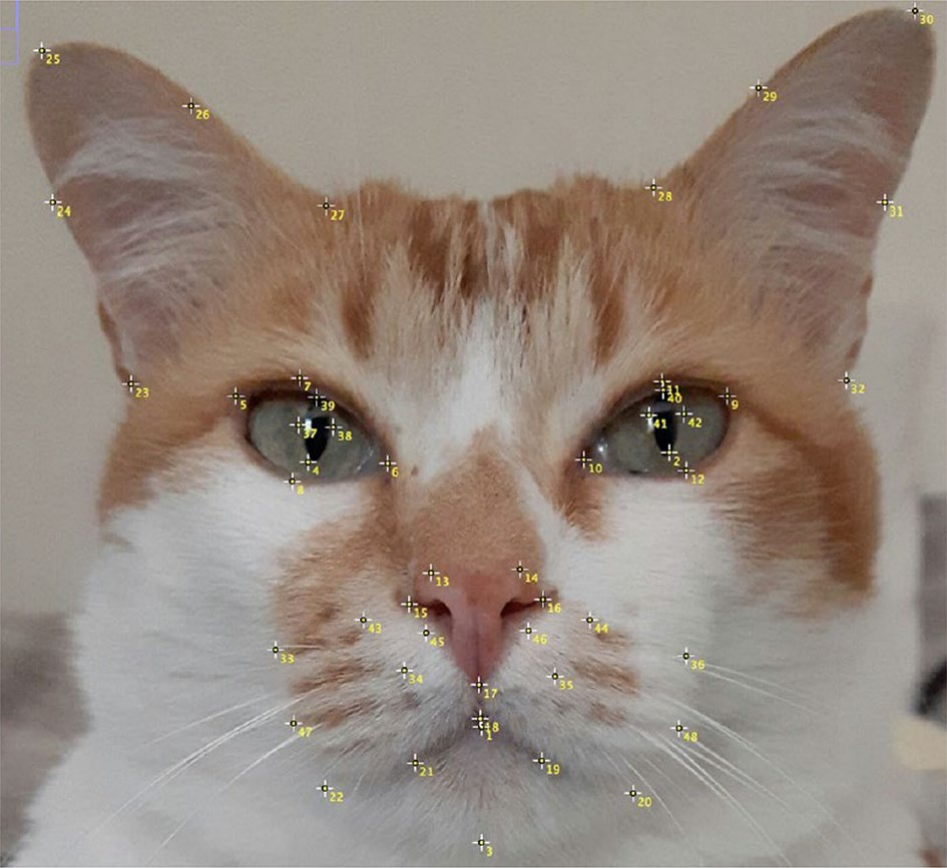
Mirror image of a cat’s face, depicting placement of the 48 facial landmarks from Finka et al.^26^. Landmarks appear contralateral to their origin, as they would when directly observing the cat’s face.

For the LDM approach, the images were manually annotated with 48 landmarks, following the approach in Finka et al. and Feighelstein et al.^26,27^, which were specifically chosen for their relationship with underlying musculature, and relevance to cat-specific facial Action Units (catFACS^35^). For the specific location of each landmark, see Fig. 3.

**Table 3:** Performance comparison between landmark-based (LDM) and deep learning (DL) approaches; bold shows the best accuracy in both approaches.

Approach	Align	Augment	Model	Accuracy	Precision	Recall
LDM	No	No	MPL	0.6806	0.7071	0.7
	No	No	RF (Max Depth: 7; Trees: 61)	0.7152	0.7898	0.73
	No	Yes (Rep=10, M=0.1)	MPL	0.7166	0.8121	0.585
	No	Yes (Rep=10,M=0.1)	RF (Max Depth: 20; Trees: 221)	0.775	0.7583	0.835
	Yes	No	MPL	0.6639	0.7121	0.685
	Yes	No	RF (Max Depth: 4 ; Trees: 121 )	**0.7611**	0.7848	0.775
	Yes	Yes (Rep=10,M=0.05)	MPL	0.6861	0.7317	0.575
	Yes	Yes (Rep=10,M=0.05)	RF (Max Depth: 1; Trees: 141)	0.7138	0.755	0.725
DL	No	No	ResNet50	**0.6514**	0.6726	0.715
	No	Yes		0.5694	0.6005	0.55
	Yes	No		0.6361	0.6414	0.635
	Yes	Yes		0.5917	0.7267	0.435

### Model performance

For measuring performance of models, we use standard evaluation metrics of accuracy, precision, recall (see, e.g., Lencioni et al^36^ for further details). As a validation method^37^, we use 10-fold cross validation with no subject overlap. This method is recommended^38^ whenever the dataset contains no more than one sample of each individual.

Table 3 presents the results of the comparison of the performance of different models (two types for each approach), with and without alignment and augmentation, which are techniques of data pre-processing that can potentially improve performance. It can be seen that the landmark-based approach performs better, with the Random Forest (RF) model reaching accuracy above 77% in pain/no pain classification as opposed to only above 65% reached by the ResNet model.

### Facial parts importance

**Figure 4:**
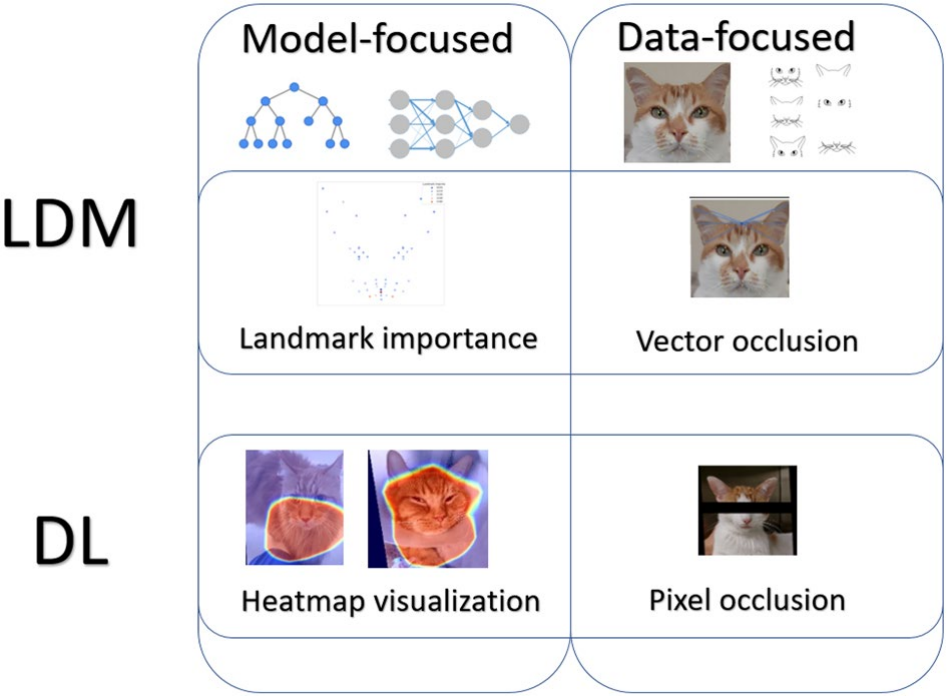
Demonstration of the different explainability approaches used in this study relevant for the landmark-based (LDM) and deep learning (DL) approaches examined.

Explainable AI methods can be roughly divided into two types^39,40^, as demonstrated on Fig. 4: data-focused and model-focused.

#### Data-focused explainability

 In this approach, the idea is to occlude information on different facial regions from the model, exploring the impact of different regional occlusions on model classification accuracy. In the context of the importance of cat facial parts, we define the following general notions for occlusion configurations for a particular face region:

- ‘Full information’: the model is trained and tested using information from all regions; *R*
- ‘Reveal only R’: the model is trained and tested on information f from only one specific region; *R*
- ‘Hide R’: the model is trained and tested on information from all regions, excluding one specific region.

**Figure 5:**
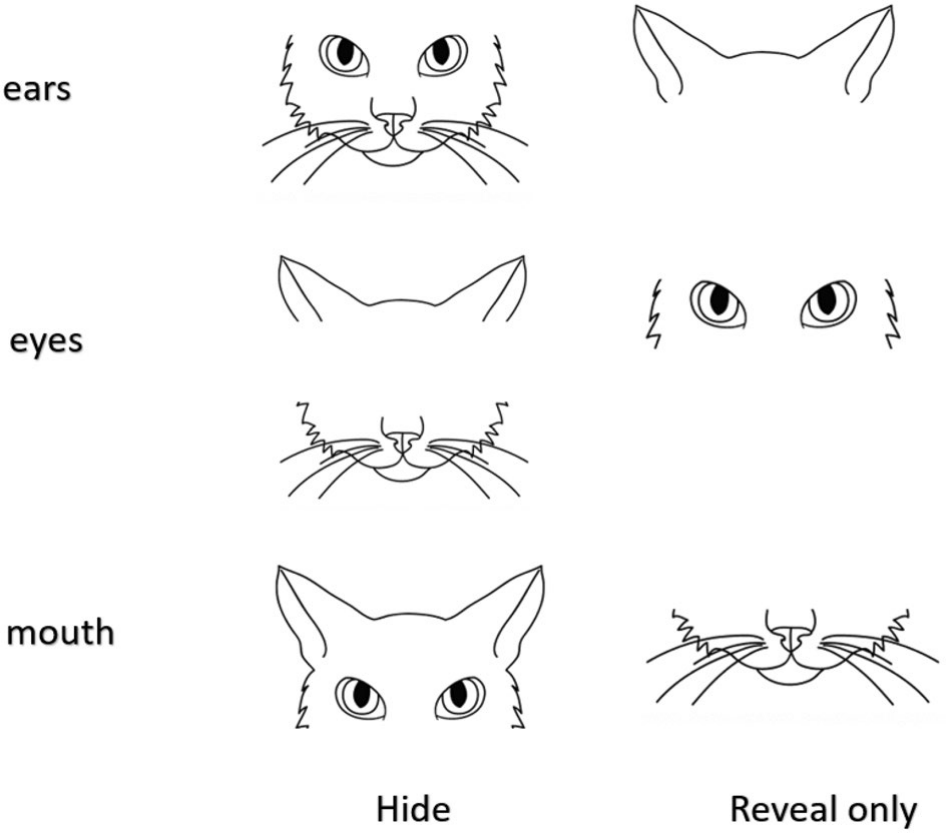
Occlusion configurations hide/reveal only for ears, eyes and mouth regions of the cat face.

Figure 5 demonstrates the occlusion configurations for each of the three regions (ears, eyes, mouth). It should be noted, however, that the relationships between the accuracies in the two configurations are not linear: having a good performance in a model exposed only to ears, does not necessarily imply having low performance when exposed to eyes and mouth only. Another thing that should be noted is that if we extend the notion of these configurations from single region to sets of regions, then there is a direct link between the two configurations: e.g., ‘hide’ configuration for eyes is equivalent to ‘reveal only’ configuration for ears and mouth. It should also be noted that in the LDM approach the input to the model is derived from manually annotated landmark information based on XY coordinates, while in the DL approach it is raw image pixel-based information. Thus the “occlusion” processes applied to these two different models are performed on different units of information.

*The LDM approach* The units of information here are vectors (ordered pairs of (x,y) coordinates of the landmarks) in different facial regions, and ‘occlusion’ is achieved by excluding vectors belonging to a certain facial region. Tables 4 and 5 present the classification results using different occlusion configurations for the Random Forest and MPL classifiers respectively. There is agreement between the two classifiers that hiding ears gives very good (roughly as “all”) accuracy, while using only ears has low accuracy. Moreover, there is also agreement that using only mouth gives good accuracy, while by hiding mouth accuracy drops (compared to “all”).

*The DL approach* The units of information here are raw pixels, and ‘occlusion’ is achieved by hiding different combinations of face mask regions (ears, eyes and mouth). As the dataset is aligned, having all eye centers located on same image position, we identify the eye mask area for all images as the area captured between the minimal and maximal y coordinate of any eye landmark. The ear mask region starts at the upper border of the image and ends at the top of the eye region. The mouth mask region starts at the bottom of the eye band and ends on the bottom of the image. We decided to use general masks for all the images instead of tailoring different regional masks per image according to their landmarks, in order to prevent that the deep learning model will obtain any information from the particular location of the tailored masks.

Table 6 presents the classification results using different occlusion configurations. As in the LDM approach, in DL hiding ears still gives good (relative to “all”) accuracy, while using only ears has lower accuracy. Moreover, using only mouth also gives good (relative to “all”) accuracy, while by hiding mouth accuracy drops (compared to “all”).

**Table 4:** Occlusion study: mirror image, LDM approach, Random forest, not aligned, augmented, depth=20, trees= 221.

Region	Config	Visualization	Accuracy	Precision	Recall
All			0.7722	0.763	0.805
Ears	Reveal only		0.412	0.442	0.385
	Hide		0.75	0.7716	0.775
Eyes	Reveal only		0.7013	0.7225	0.755
	Hide		0.6903	0.7333	0.66
Mouth	Reveal only		0.7167	0.7366	0.695
	Hide		0.6722	0.6633	0.715

**Table 5:** Occlusion study: LDM approach, MPL, not aligned, augmented.

Region	Config	Visualization	Accuracy	Precision	Recall
All			0.6933	0.7565	0.607
Ears	Reveal only		0.4309	0.3832	0.2925
	Hide		0.7055	0.7683	0.5955
Eyes	Reveal only		0.6154	0.6314	0.541
	Hide		0.6677	0.7508	0.5555
Mouth	Reveal only		0.7183	0.7883	0.6225
	Hide		0.6027	0.6581	0.498

**Table 6:** Occlusion study - DL approach.

Region	Config	Visualization	Accuracy	Precision	Recall
All			0.6361	0.6414	0.635
Ears	Reveal only		0.6083	0.655	0.53
	Hide		0.6153	0.6483	0.495
Eyes	Reveal only		0.525	0.5829	0.556
	Hide		0.5989	0.6539	0.5996
Mouth	Reveal only		0.6107	0.7067	0.4004
	Hide		0.5708	0.6571	0.535

### Model-focused explainability

These methods are based on extracting information from the model itself, e.g. information on feature relevance such as using back-propagation algorithms in neural networks, or feature importance rating in tree-based models.

**Figure 6:**
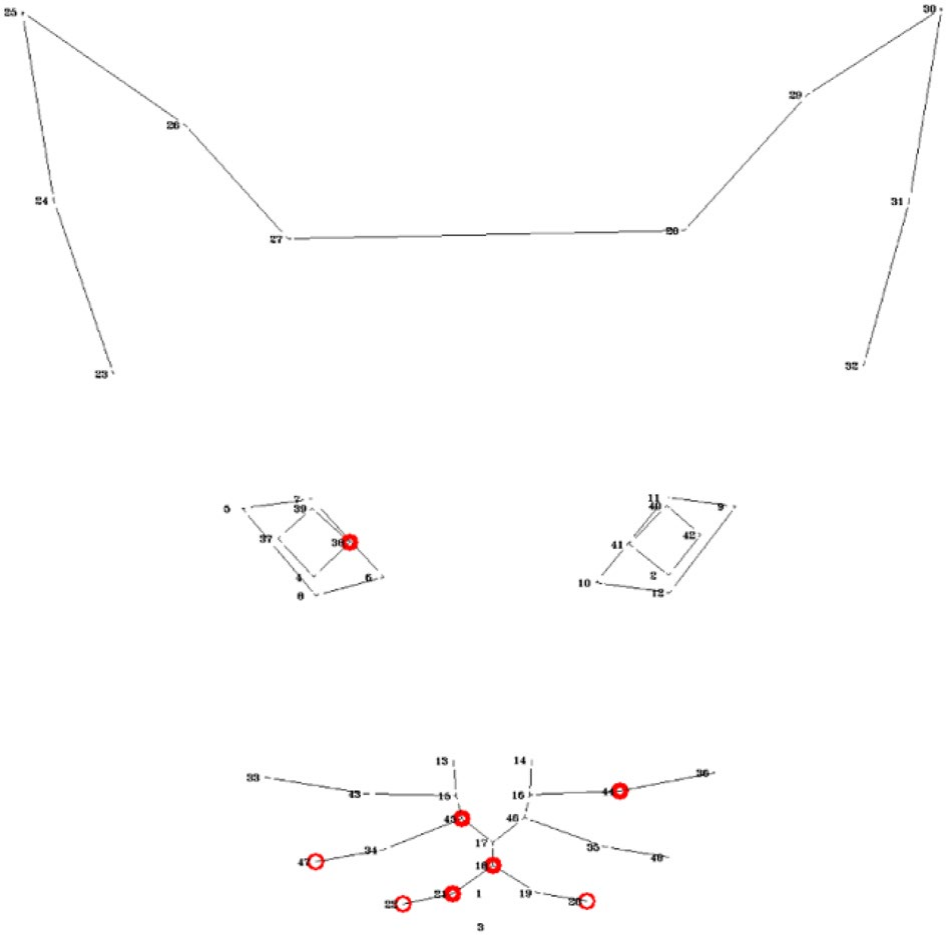
LDM approach, Random Forest. Landmark importance was min-max normalized to values between 0 and 1. A landmark appears in red if its relative importance is greater or equal to 0.5.

*The LDM approach* In this approach the use of Random Forest models allows for extracting information on feature importance^41^ for each of the landmarks. More specifically, we utilize the Gini Importance or Mean Decrease in Impurity (MDI) metric^42^ that calculates each feature importance as the sum over the number of splits (accross all trees) that include the feature, proportionate to the number of samples it splits^43^. Once the model is trained, we calculate the individual landmark importance as the sum of the feature importance of its input coordinates x and y. Figure 6 presents the feature importance of all the 48 landmarks (aggregated over all images), with red colors indicating more important landmarks and the deepness of the red color reflecting relative importance, with the majority of most important landmarks appearing in the mouth area.

**Figure 7:**
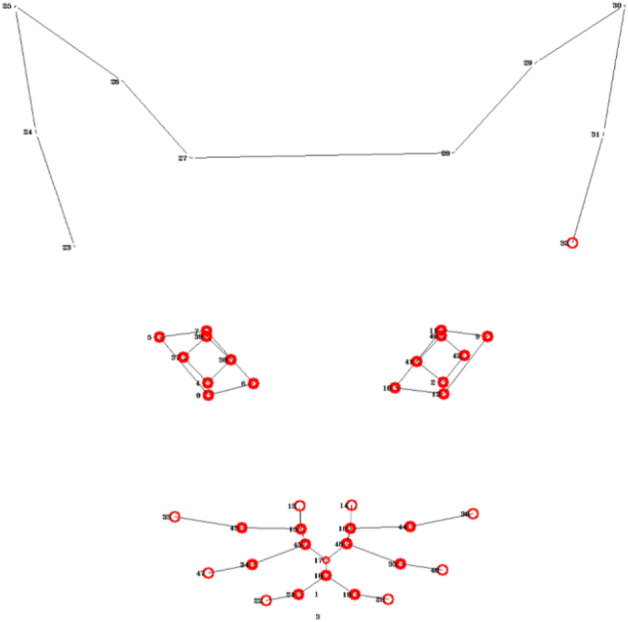
DL approach, mirror image. Average heat for landmarks, min-max normalized to values between 0 and 1. A landmark appears in red if its relative importance is greater or equal to 0.5.

**Figure 8:**
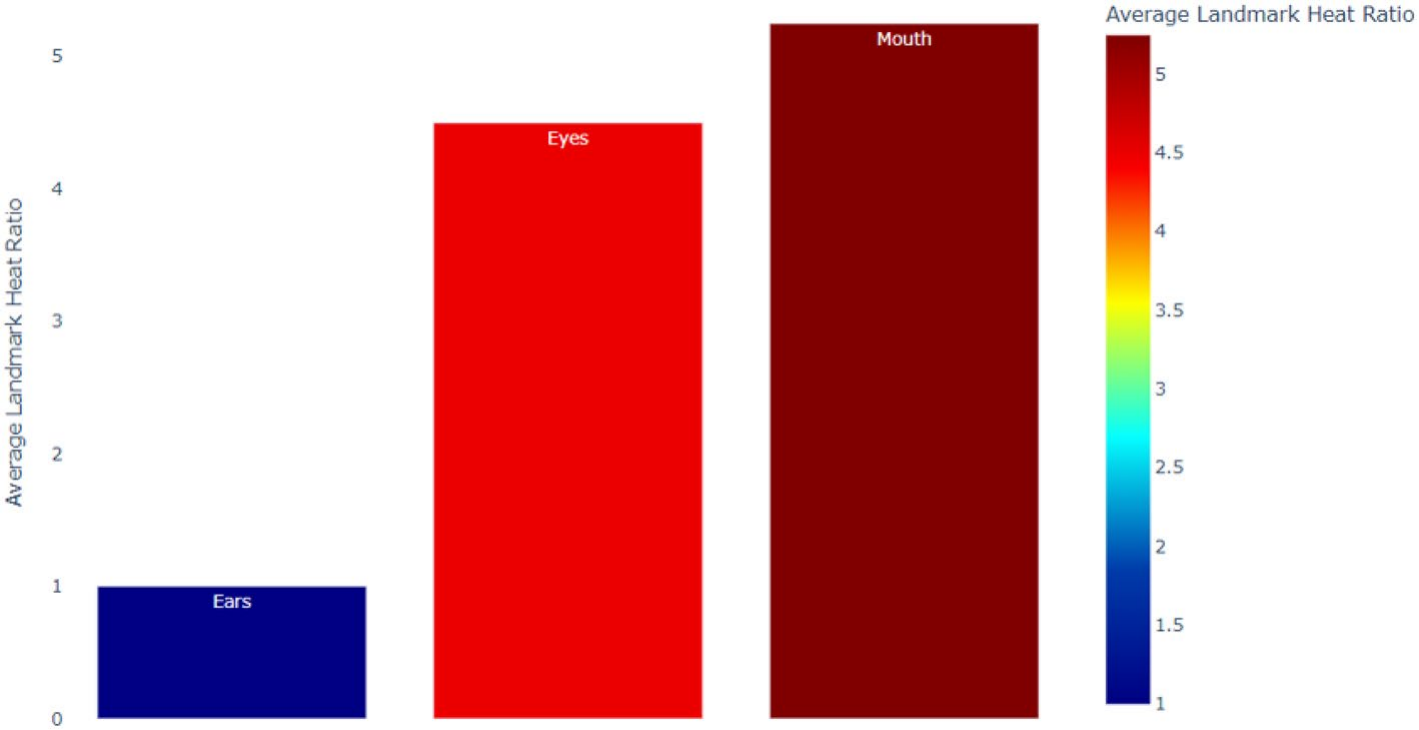
DL approach. Average heat for face parts.

*The DL approach* In the DL approach, we employ one of the most commonly used approaches is the GradCAM method^44,45^ to visualize heatmaps, showing the ‘attention’ areas of the trained ResNet50 network. The availability of landmark annotations from the LDM allows also for a more sophisticated quantitative analysis of the heatmaps, quantifying the degree of attention (heat) of the model per landmark (Fig. 7) and per face region (Fig. 8). This shows mouth and eyes are clearly more “informative” for the classifer than ears.

Table 9 presents a summary of indications consistent across both LDM and DL approaches, showing that the mouth is most important, and ear are the least important facial part for the classifiers.

**Figure 9:**
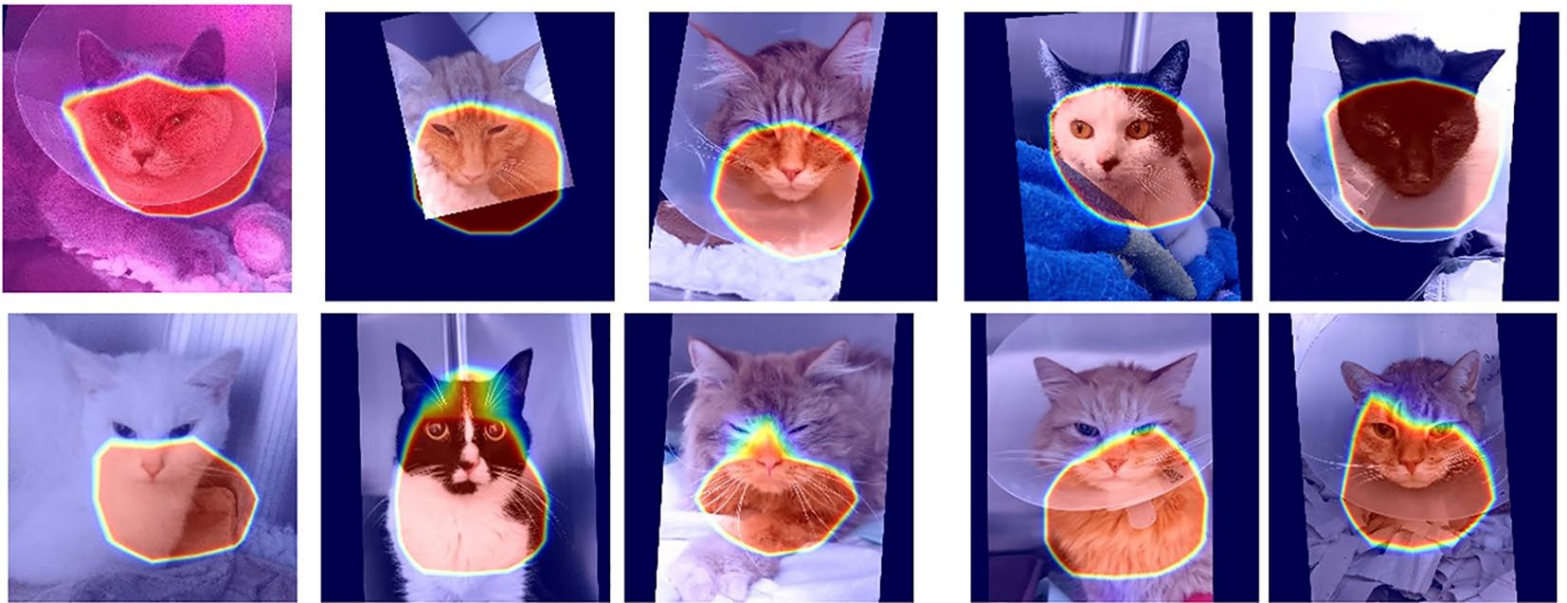
Example heatmaps. Top: correctly classified as ’pain’.; Bottom: correctly classified as ’no pain’.

**Figure 10:**
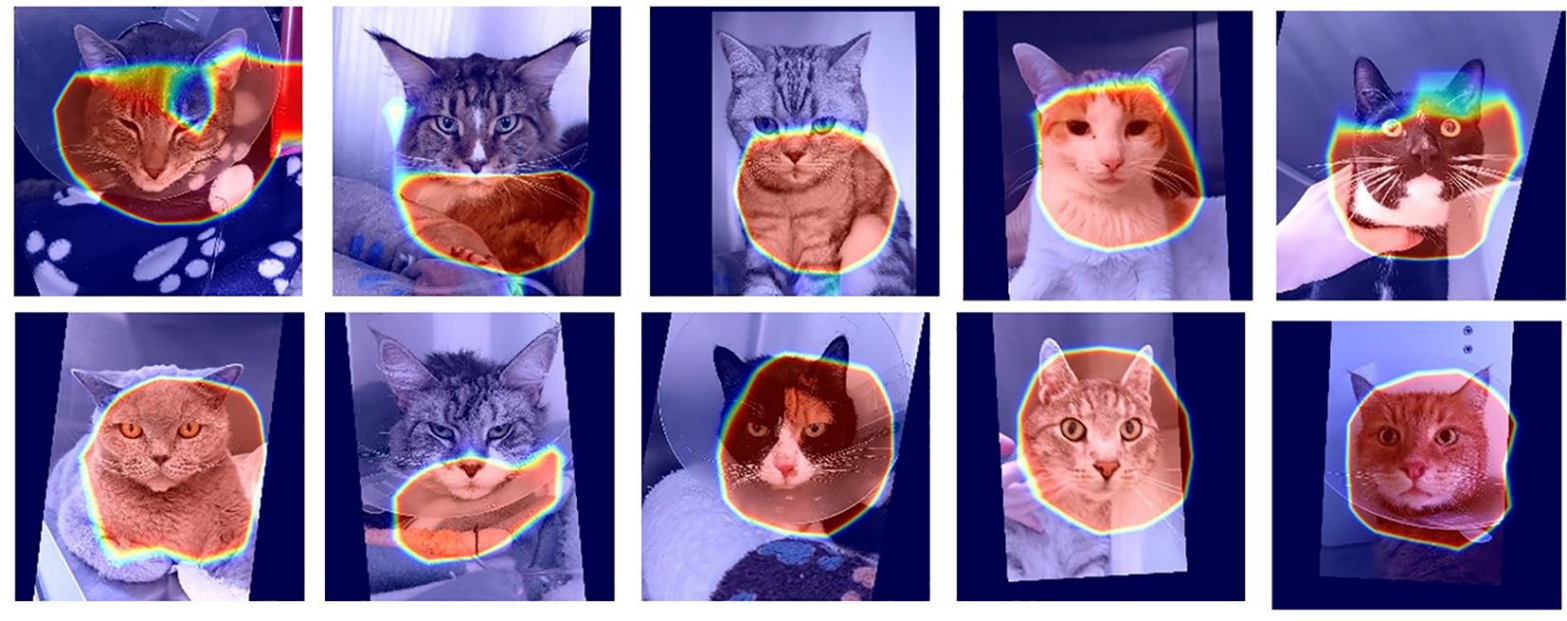
Example heatmaps. Top: incorrectly classified as ’pain’.; Bottom: incorrectly classified as ’no pain’.

Figs. 9 and 10 present examples of GradCAM heatmaps extracted from images within our dataset. The hotter (deeper red) the pixel appears to be in a heatmap, the more attention is given to it by the model for pain/no pain classification. The colder (more blue) pixels are those receiving less attention from the model.

**Table 7:** Summary of data-driven explainability results.

Approach		Reveal only		Hide	
		Highest	Lowest	Highest	Lowest
LDM	RF	Mouth	ears	Ears	Mouth
	MPL	Mouth	ears	Ears	Mouth
DL	ResNet	Mouth	eyes	Ears	Mouth

**Table 8:** Average landmark heat/feature importance ratio per region - ResNet50 and random forest.

Face region	Avg. heat (ResNet - DL)	Avg. importance (Random forest - LDM)
Ears	1.00	1.00
Eyes	3.48	1.17
Mouth	3.97	2.07

**Table 9:** Summary of model-driven explainability results.

Approach		Highest	Lowest
LDM	Landmark feature importance	Mouth	Ears
DL	Landmark average heat	Mouth	Ears

## Discussion

Feighelstein et al.^27^ showed that the LDM and DL approaches performed comparably well on a single-breed, single-sex, single condition data set, with both models reaching accuracy above 72%. The current study provides further indication for the success of the LDM approach, reaching an improved performance rate of above 77% on a more heterogeneous data population. The DL approach, on the other hand, is less successful on this more diverse dataset, reaching only around 65% accuracy. This drop in performance of the DL approach is however most likely due to the current dataset being much smaller than that of Feighelstein et al.^27^ (464 images in the previous study as opposed to 84 here), given that deep learning approaches tend to be data-hungry. Thus investigating whether the performance of the DL approach is improved by enlarging the dataset is an immediate priority for future research. Landmark-based approaches are by their nature better able to directly measure and thus better account for variability in morphology of the cat faces (as opposed to DL approaches which use raw pixel data and may be “confused” by this variability), which could explain their robustness on this dataset. Another important difference between the study of Feighelstein et al.^27^ and this study is the ground truth labelling of pain/no pain classes. Broomé et al.^38^ reviews labelling methods in the context of automated recognition of animal affect and pain, dividing into two main ways: behavior-based or stimulus-based state annotations. The former are purely based on the observed behaviors, and are usually scored by human experts. For the latter, the ground-truth is based on whether the data were recorded during an ongoing stimulus or not. In Feighelstein et al.^27^, the time points when the images were taken provide a stimulus-based method, as the participant’s images were captured after ovariohysterectomy at different time points corresponding to varying (controlled) intensities of pain (i.e. pre or post op and pre and post rescue analgesia). In the current study however, images of cats’ faces were recorded in a real-life veterinary context where pain was naturally occurring rather than clinically induced/controlled and ‘pain/no pain’ labelling was derived from a subsequently conducted behavior-based assessment method, (the CMPS-feline^23^, based on real-time human-inferences of cat behavioural elements and facial changes.

On the more technical side, in the current study augmentation did not significantly improve model performance, which is in line with the findings in Feighelstein et al.^27^. Using Random Forest as a base model improved performance as compared to using MPL in the LDM approach. The use of multi-region vectorization led to improved performance in the LDM approach. The vectors were defined based on the cat face regions as defined by the FGS^15^, and thus they seem to “guide” the model in “looking” within each region separately, without linking anatomically unrelated landmarks. In this way the vectors can be efficient in holistically capturing the outputs of subtle differences in the relative positioning of underlying facial musculature that may occur as a consequence of the micromovements of the muscle contractions in cats’ faces. Vector based approaches thus provide a more efficient geometric morphometric representation of the cat face for pain recognition than just using the set of landmarks with no connections between them.

To summarize, our first findings suggest that in relation to pain/no pain discrimination accuracy, the annotation approach using landmarks is potentially more robust for use on noiser more naturalistic populations and where resulting datasets are of a modest size. However, the downside of taking this route is the resource and effort needed for landmark annotation given this is currently required to be completed manually. Thus one natural direction for future research is the automation of detection and annotation of cat facial landmarks. While a vast body of work addresses this problem for human faces (see Wu and Ji^46^ for a review), the topic of landmark localization for animal faces is currently understudied. Development of such methods for cats will provide an essential step toward accurate automated cat pain recognition in clinical and other practical settings and may pave the way for subsequent cross-species application.

A further important finding of this study is summarized in Table 9, showing a striking consistency across approaches (LDM vs. DL, RF vs. MPL) with respect to occlusion experiments: using only information on the ears leads to low performance, while using only information on the mouth still delivers high performance. Moreover, hiding ears improves performance, while hiding the mouth decreases performance. This is further strengthened by the feature importance information extracted both in LDM and DL approaches (Table 7): features related to the ears appear to be the least important, while features related to the mouth appear to be the most important in both cases.

While a possible interpretation of this finding might be that the cat’s mouth is more expressive than other facial regions, in Evangelista et al.^15^ the cat’s ears were reported as a more reliable visual indicator during human FGS scoring compared to the eyes (i.e., the ears had better internal consistency). Thus, an alternative and more probable explanation could be that the mixed-breed dataset used in the current study introduced greater baseline noise concerning the general shape and size of ears (i.e., Finka et al.^29^) than could be handled by the machine learning approaches in order to use these features to reliably classify images based on pain presence/absence. However, low performance of the ears could also be attributed to other features associated with the specific dataset used in this study such as the way images were collected (i.e. the angle of the camera relative to the cat, or lighting conditions etc). The potential impact of such factors should be investigated in future studies. Another point worth noting is that this finding could also be related to the static (image-based) analysis performed in this study; in future investigations it should be checked whether it is also preserved in video-based approaches. One immediate research priority is therefore to investigate whether it is indeed the case that the machine “sees” pain differently to humans. One way to proceed would be to compare machine classification to human expert performance using methods such as face masking, similar to the idea used in the works^47–49^.

A limitation of the current study that should be mentioned is the size of the dataset used, as well as a majority of male (two thirds) cats in it. Another limitation is the use of photos, which capture just one momentary facial expression. As already mentioned above, the use of video data in the development of AI models can enable the analysis of both facial expressions and behavioral indicators of pain by taking into account the temporal dimension. As such approaches tend to be significantly data-hungry^38^, expanding the available datasets on cat pain from images to videos should be a priority for the development of AI models suitable for clinical settings.

The results presented in this study further support the indication from Feighelstein et al.^27^ that AI-assisted recognition of negative affective states such as pain from cat faces is feasible.

However, negative affective states can also be associated with other distressful conditions (e.g., anaemia, nausea). In order to differentiate pain from these conditions, further data acquisition with appropriate diagnostics is necessary. For this reason, the correlation of sampled footage with the corresponding clinical records is essential for the development of clinically supportive and multifaceted tools to differentiate painful and non-painful conditions causing a negative affective state. Due to the lack of verbal communication in animals, further development and optimization of these tools can be an important contribution to the adequate treatment of pain in cats. For this purpose, further data are necessary in order to guarantee appropriate generalizability of automated pain recognition especially among different cat breeds, medical conditions, technical possibilities, and environments. However, AI systems should be seen as a complement to and not a replacement of clinical judgement skills, with the potential to increase awareness of cases requiring greater attention and care.

## Methods

### Reliability of annotation

To establish the reliability of the landmark annotation process, a second person manually annotated more than 10% of images from the dataset, using the same annotation instructions. Images used for reliability analysis were selected pseudo randomly, so that contributions were balanced across individuals and conditions. At the point of annotation, both annotators were blinded to the condition from which each image was drawn. Inter-annotator reliability for the 96 XY coordinates was determined via the Inter Class Correlation Coefficient ICC2 (a measure of absolute agreement between raters^50^), and reached the threshold for ICC2 acceptability.

### Model training

- *DL* The approach was as per Feighelstein et al.^27^, we apply transfer learning on a Resnet50 model pre-trained on ImageNet, adding a new sub network compound on top of the last layer with the parameters specified in this study^27^.
- *LDM* The approach was as per Feighelstein et al.^27^, we trained a Multi Layer Perceptron neural network (MLP), consisting of an input layer containing 96 neurons (one for each x and y coordinate obtained via the 48 landmarks) with the parameters specified in this study^27^. Additionally, due to its supporting feature importance extraction^41^, we trained also a Random Forest model, optimizing accuracy while ranging maximal depth (*MaxDepth*) of trees between 1 and 40 and number of estimators (*Trees*) from 1 to 250 in intervals of 5. Optimal parameters *MaxDepth* and *Trees* for each input configuration are specified in Table 3.

### Average heat calculation

To calculate the average heat per face region (see Table 8 and Fig. 8), we took advantage of the availability of landmark annotations of the dataset. We calculate for all cat face images the average heat of every landmark on their corresponding heatmaps. More formally, let *I* be an image and *I*(*x*, *y*) - the pixel of *I* with coordinates (*x*, *y*). Denote by (*R*, *G*, *B*)[*p*] the (R,G,B) color component of pixel *p*. Denote by $(x^I_L,y^I_L)$ the coordinates of landmark *L* on image *I*. Then the heat of *L* on *I* is defined by $Heat(L,I)=(R,G,B)[p]$, where $p = I(x^I_L,y^I_L)$. The average heat of a landmark *L* is obtained by averaging over all *I* in the dataset. We then normalize this by dividing the result by the maximal value of the color components of all landmark and multiplying by 255. To compare heat across face regions, we further aggregated the average heat over each region.

## Data Availability

The dataset is available from the corresponding authors upon request.

## References

[1] Raja SN (2020). The revised IASP definition of pain: Concepts, challenges, and compromises. Pain.

[2] Lichtner V (2014). Pain assessment for people with dementia: A systematic review of systematic reviews of pain assessment tools. BMC Geriatr..

[3] Lascelles B (2019). Measurement of chronic pain in companion animals: Discussions from the pain in animals workshop (paw) 2017. Vet. J..

[4] Weber G, Morton J, Keates H (2012). Postoperative pain and perioperative analgesic administration in dogs: practices, attitudes and beliefs of queensland veterinarians. Australian Veterinary Journal.

[5] Williams V, Lascelles B, Robson M (2005). Current attitudes to, and use of, peri-operative analgesia in dogs and cats by veterinarians in new Zealand. New Z. Vet. J..

[6] Bell A, Helm J, Reid J (2014). Veterinarians’ attitudes to chronic pain in dogs. Vet. Rec..

[7] Mogil JS, Pang DS, Dutra GGS, Chambers CT (2020). The development and use of facial grimace scales for pain measurement in animals. Neurosci. Biobehav. Rev..

[8] Sotocina SG (2011). The rat grimace scale: A partially automated method for quantifying pain in the laboratory rat via facial expressions. Mol. Pain.

[9] Keating SC, Thomas AA, Flecknell PA, Leach MC (2012). Evaluation of EMLA cream for preventing pain during tattooing of rabbits: changes in physiological, behavioural and facial expression responses.

[10] Dalla Costa E (2014). Development of the horse grimace scale (HGS) as a pain assessment tool in horses undergoing routine castration. PLoS One.

[11] Di Giminiani P (2016). The assessment of facial expressions in piglets undergoing tail docking and castration: toward the development of the piglet grimace scale. Front. Vet. Sci..

[12] McLennan KM (2016). Development of a facial expression scale using footrot and mastitis as models of pain in sheep. Appl. Anim. Behav. Sci..

[13] Reijgwart ML (2017). The composition and initial evaluation of a grimace scale in ferrets after surgical implantation of a telemetry probe. PloS One.

[14] Holden E (2014). Evaluation of facial expression in acute pain in cats. J. Small Anim. Pract..

[15] Evangelista MC (2019). Facial expressions of pain in cats: The development and validation of a feline grimace scale. Sci. Rep..

[16] Lascelles BDX, Robertson SA (2010). Djd-associated pain in cats: What can we do to promote patient comfort?. J. Feline Med. Surg..

[17] Merola I, Mills DS (2016). Behavioural signs of pain in cats: An expert consensus. PloS One.

[18] Dawson, L., Niel, L., Cheal, J. & Mason, G. *Humans can identify cats’ affective states from subtle facial expressions* (UFAW, 2019).

[19] Hunt JR, Knowles TG, Lascelles B, Murrell JC (2015). Prescription of perioperative analgesics by UK small animal veterinary surgeons in 2013. Vet. Rec..

[20] Hewson CJ, Dohoo IR, Lemke KA (2006). Factors affecting the use of postincisional analgesics in dogs and cats by Canadian veterinarians in 2001. Can. Vet. J..

[21] Watson A, Nicholson A, Church D, Pearson M (1996). Use of anti-inflammatory and analgesic drugs in dogs and cats. Aust. Vet. J..

[22] Brondani JT (2013). Validation of the english version of the UNESP-BOTUCATU multidimensional composite pain scale for assessing postoperative pain in cats. BMC Vet. Re..

[23] Reid, J., Scott, E., Calvo, G. & Nolan, A. Definitive Glasgow acute pain scale for cats: validation and intervention level. *Vet. Rec.* **108** (2017). 10.1136/vr.104208 28130405

[24] Evangelista MC (2020). Clinical applicability of the feline grimace scale: Real-time versus image scoring and the influence of sedation and surgery. PeerJ.

[25] Evangelista MC, Steagall PV (2021). Agreement and reliability of the feline grimace scale among cat owners, veterinarians, veterinary students and nurses. Sci. Rep..

[26] Finka LR (2019). Geometric morphometrics for the study of facial expressions in non-human animals, using the domestic cat as an exemplar. Sci. Rep..

[27] Feighelstein M (2022). Automated recognition of pain in cats. Sci. Rep..

[28] Künzel W, Breit S, Oppel M (2003). Morphometric investigations of breed-specific features in feline skulls and considerations on their functional implications. Anat. Histol. Embryol..

[29] Finka LR, Luna SP, Mills DS, Farnworth MJ (2020). The application of geometric morphometrics to explore potential impacts of anthropocentric selection on animals’ ability to communicate via the face: The domestic cat as a case study. Front. Vet. Sci..

[30] Fleming PA, Crawford HM, Auckland C, Calver MC (2021). Nine ways to score nine lives-identifying appropriate methods to age domestic cats ( Felis Catus). J. Zool..

[31] Schmidt MJ (2022). Closure times of neurocranial sutures and synchondroses in Persian compared to domestic shorthair cats. Sci. Rep..

[32] Pitakarnnop T, Buddhachat K, Euppayo T, Kriangwanich W, Nganvongpanit K (2017). Feline (Felis Catus) skull and pelvic morphology and morphometry: Gender-related difference?. Anat. Histol. Embryol..

[33] Quinn PC, Palmer V, Slater AM (1999). Identification of gender in domestic-cat faces with and without training: Perceptual learning of a natural categorization task. Perception.

[34] Minh D, Wang HX, Li YF, Nguyen TN (2021). Explainable artificial intelligence: A comprehensive review. Artif. Intell. Rev..

[35] Caeiro CC, Burrows AM, Waller BM (2017). Development and application of Catfacs: Are human cat adopters influenced by cat facial expressions?. Appl. Anim. Behav. Sci..

[36] Lencioni GC, de Sousa RV, de Souza Sardinha EJ, Corrêa RR, Zanella AJ (2021). Pain assessment in horses using automatic facial expression recognition through deep learning-based modeling. PloS One.

[37] Refaeilzadeh P, Tang L, Liu H (2009). Cross-Validation.

[38] Broomé, S. *et al.* Going deeper than tracking: A survey of computer-vision based recognition of animal pain and affective states. arXiv preprint arXiv:2206.08405 (2022).

[39] Linardatos P, Papastefanopoulos V, Kotsiantis S (2020). Explainable ai: A review of machine learning interpretability methods. Entropy.

[40] Das, A. & Rad, P. Opportunities and challenges in explainable artificial intelligence (xai): A survey. arXiv preprint arXiv:2006.11371 (2020).

[41] Louppe, G. *Understanding Random Forests: From Theory to Practice*. Ph.D. thesis (2014). 10.13140/2.1.1570.5928.

[42] Han, H., Guo, X. & Yu, H. Variable selection using mean decrease accuracy and mean decrease gini based on random forest. In *2016 7th IEEE International Conference on Software Engineering and Service Science (ICSESS)*, 219–224 (IEEE, 2016).

[43] Breiman L (2001). Random forests. Mach. Learn..

[44] Selvaraju, R. R. *et al.* Grad-cam: Visual explanations from deep networks via gradient-based localization. In *Proceedings of the IEEE International Conference on Computer Vision*, 618–626 (2017).

[45] Selvaraju, R. R. *et al.* Grad-cam: Visual explanations from deep networks via gradient-based localization. *Int. J. Comput. Vis.* **128**(2) (2020).

[46] Wu Y, Ji Q (2019). Facial landmark detection: A literature survey. Int. J. Comput. Vis..

[47] Smith ML, Cottrell GW, Gosselin F, Schyns PG (2005). Transmitting and decoding facial expressions. Psychol. Sci..

[48] Gosselin F, Schyns PG (2001). Bubbles: A technique to reveal the use of information in recognition tasks. Vis. Res..

[49] Wegrzyn M, Vogt M, Kireclioglu B, Schneider J, Kissler J (2017). Mapping the emotional face. How individual face parts contribute to successful emotion recognition. PloS One.

[50] Shrout PE, Fleiss JL (1979). Intraclass correlations: Uses in assessing rater reliability. Psychol. Bull..

